# A retrospective, naturalistic study of deep brain stimulation and vagal nerve stimulation in young patients*

**DOI:** 10.1002/brb3.3452

**Published:** 2024-03-11

**Authors:** Deniz Yuruk, Can Ozger, Juan F. Garzon, Paul A. Nakonezny, Jennifer L. Vande Voort, Paul E. Croarkin

**Affiliations:** ^1^ Research Fellow in the Department of Psychiatry and Psychology Mayo Clinic School of Graduate Medical Education, Mayo Clinic College of Medicine and Science Rochester Minnesota USA; ^2^ Department of Psychiatry and Psychology Mayo Clinic Children's Research Center, and Mayo Clinic Depression Center, Mayo Clinic Rochester Minnesota USA; ^3^ Department Of Population And Data Sciences UT Southwestern Medical Center Dallas Texas USA

**Keywords:** adolescent psychology, neuropsychiatrics, neurosurgery, psychiatry

## Abstract

**Introduction:**

Invasive neuromodulation interventions such as deep brain stimulation (DBS) and vagal nerve stimulation (VNS) are important treatments for movement disorders and epilepsy, but literature focused on young patients treated with DBS and VNS is limited. This retrospective study aimed to examine naturalistic outcomes of VNS and DBS treatment of epilepsy and dystonia in children, adolescents, and young adults.

**Methods:**

We retrospectively assessed patient demographic and outcome data that were obtained from electronic health records. Two researchers used the Clinical Global Impression scale to retrospectively rate the severity of neurologic and psychiatric symptoms before and after patients underwent surgery to implant DBS electrodes or a VNS device. Descriptive and inferential statistics were used to examine clinical effects.

**Results:**

Data from 73 patients were evaluated. Neurologic symptoms improved for patients treated with DBS and VNS (*p* < .001). Patients treated with DBS did not have a change in psychiatric symptoms, whereas psychiatric symptoms worsened for patients treated with VNS (*p* = .008). The frequency of postoperative complications did not differ between VNS and DBS groups.

**Conclusion:**

Young patients may have distinct vulnerabilities for increased psychiatric symptoms during treatment with invasive neuromodulation. Child and adolescent psychiatrists should consider a more proactive approach and greater engagement with DBS and VNS teams that treat younger patients.

## Graphical abstract

Deep brain stimulation (DBS) and vagal nerve stimulation (VNS) are increasingly used to treat movement disorders and epilepsy in pediatric patients and young adults. Previous research showed that psychiatric comorbid conditions also are common in patients with these conditions. Much prior research was conducted regarding the short‐term effects of DBS and VNS, but the clinical outcomes of young patients, months and years after DBS and VNS treatment, are unclear. Therefore, we aimed to examine the neurologic and psychiatric outcomes of DBS and VNS in children, adolescents, and young adults. We examined data from 73 patients who were treated with these modalities and compared their symptoms before and after the procedure. Our results suggested that neurologic symptoms improved with DBS and VNS, psychiatric symptoms did not change in patients treated with DBS, but psychiatric symptoms worsened for patients treated with VNS. We conclude that young patients treated with DBS and VNS may be vulnerable to increased psychiatric symptoms. A collaborative treatment model that involves child and adolescent psychiatrists working with DBS and VNS teams should be considered for younger patients.

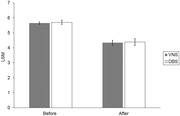

## 1 LIMITATIONS

This study had notable limitations due to its retrospective, naturalistic design and its reliance on information from healthcare provider notes. We were not able to account for differences in electrode placement with respect to DBS. Documentation was likely inconsistent within the sample. The availability of information needed to assign CGI scores varied from patient to patient. The sample was also heterogeneous with respect to age, symptoms, neurologic diagnoses, and psychiatric diagnoses. In addition, most patients had multiple psychiatric comorbidities, and their psychiatric CGI scores were based on the total severity of symptoms without discriminating among diagnoses. Although the CGI is useful for representing the psychiatric symptom severity of patients, it is not possible to make conclusions regarding specific disorders. Similarly, if one cluster of symptoms reduced while another increased, this change would not be detected. Our findings should be considered in the context of these inherent limitations. This initial work serves an important role in hypothesis generation for future, rigorous, and prospective studies of VNS and DBS that focus on younger patients and their psychiatric outcomes.

## 2 INTRODUCTION

Invasive neuromodulation treatment involves the surgical implantation of electrodes and a pulse generator. Vagal nerve stimulation (VNS) electrically stimulates the tenth cranial nerve and provides afferent stimulation to the nucleus tractus solitarius. It is primarily used to treat patients with intractable epilepsy and treatment‐resistant depression (TRD) (Edwards et al., 2017). However, use for TRD is limited compared to intractable epilepsy. Deep brain stimulation (DBS), a technique that emerged in the 1960s, is used to treat several neurologic and psychiatric disorders. For DBS, leads are placed in targeted areas of the brain and are connected to a pulse generator that is implanted extracranially. As of 2022, US Food and Drug Administration−approved neuromodulation treatments for children are limited to VNS for treatment‐resistant epilepsy and DBS for dystonia.

Psychiatric comorbidities are common with neurologic disorders (Austin et al., 2001; Hesdorffer, 2016) and occur markedly more frequently among people with seizures than the general population (Brodtkorb & Mula, 2006). Depression and anxiety are the most common psychiatric comorbidities in patients with epilepsy; depression affects about 30% of patients, and anxiety affects 10%–25% of patients (Gaitatzis et al., 2004). A population‐based study reported a 27% prevalence of psychiatric conditions for school‐aged children with epilepsy, whereas prevalence was 7% for the general population (“A Neuropsychiatric Study in Childhood”, 1971). Similarly, another study reported that 23% of children with epilepsy were diagnosed with psychiatric disorders, compared with 8% of the general population (Hackett et al., 1998). Children with chronic epilepsy also have a markedly higher rate of behavioral problems than the general population (48% vs. 10%) (Austin et al., 2001; Camfield et al., 1993; Hoare, 1984; Lindsay et al., 1979; Shukla et al., 1979).

Previous research has compared the effectiveness of VNS and DBS. Zhu et al. (2021) evaluated patients with drug‐resistant epilepsy during 12 months of VNS or DBS. At every 3‐month follow‐up assessment, the efficacy of anterior thalamic DBS was higher than that of VNS. Additionally, a meta‐analysis comparing VNS and DBS for treatment of unipolar and bipolar depression showed that patients receiving DBS had greater improvement (Khan et al., 2018). Wong et al. (2019) examined the results of five clinical trials and showed that VNS was less efficacious than DBS over time.

Published studies have examined VNS and DBS in adult patients with psychiatric disorders. A meta‐analysis of 15 studies investigating DBS for obsessive–compulsive disorder reported that the average response rate was 60% (Alonso et al., 2015). In an open‐label trial, 17 patients with TRD were monitored after the DBS procedure, and 7 patients (41%) had remission after 12 months (Malone, 2010). Several clinical studies have shown marked improvement and remission of TRD with the use of DBS (Lozano et al., 2008, 2012; Mayberg et al., 2005). VNS can also be effective for treating adults with TRD (Sackeim et al., 2001). However, VNS and DBS have not been rigorously studied in younger patients. A randomized, double‐blind study that included 41 children with intractable seizure disorder investigated behavioral effects after implantation of a VNS device, and improvement in overall mood and the depression subscale was reported across the entire group (Klinkenberg et al., 2013). A similar study assessed 15 children after VNS treatment, and 11 showed mood improvement (Hallbook et al., 2005). Despite trials such as these, little data are available that focus on the prognosis of psychiatric comorbidities in young patients treated with DBS and VNS.

To address this knowledge gap, we conducted a naturalistic, retrospective study to compare the neurologic and psychiatric outcomes of invasive neuromodulation procedures (i.e., DBS and VNS) in children, adolescents, and young adults. We also aimed to compare the complication rates associated with these procedures. We hypothesized that both neurologic and psychiatric symptom severity would decrease after VNS and DBS procedures, with young patients undergoing DBS showing the greatest improvement. We also hypothesized that young patients undergoing DBS procedures would have more complications than patients undergoing VNS procedures.

## 3 METHODS

We conducted a retrospective study of existing clinical data. The study protocol was approved by the Mayo Clinic Institutional Review Board before any study procedures commenced. All patients completed research authorization forms, giving permission to use medical records for research.

### 3.1 Participants

We identified patients aged 25 years and younger who had DBS or VNS devices implanted for neurologic conditions from January 1, 1999, to July 1, 2019. This inclusive age range was used with the intent of examining outcomes in children, adolescents, and young adults. The upper age limit was consistent with current definitions of young adults (Simpson, 2018). Patients were identified using Advanced Cohort Explorer software (Mayo Clinic), and only those with comorbid psychiatric diagnoses were included in this study. Data were extracted from electronic health records if the record had a research authorization designation.

### 3.2 Procedures and measures

The Clinical Global Impression scale is a widely accepted, clinician‐rated instrument that is used to conduct a simplified, global assessment of illness severity (CGI‐S) and improvement (CGI‐I). The CGI‐S is a one‐item, seven‐point scale that represents the current overall severity of a patient's symptoms, and the CGI‐I is a one‐item, seven‐point scale that indicates the patient's improvement from baseline to the current date. CGI‐S and CGI‐I have been used in many clinical psychiatry studies, including numerous studies of VNS and DBS (Aaronson et al., 2017; George et al., 2008; Hilderink et al., 2017; Mayberg et al., 2005; Puigdemont et al., 2012).

In this study, the CGI was used to retrospectively assess (1) the severity of neurologic and psychiatric symptoms, before the device placement procedure and after treatment, and (2) clinical improvement with treatment. Two raters (D.Y. and J.F.G.) independently assigned CGI scores after reviewing information regarding psychiatric and neurologic symptoms that were available in the electronic health record. To assess neurologic symptom severity, they considered neurology and surgery clinician notes, dated up to 2 years before and after the surgery. To assess psychiatric symptom severity, they considered psychiatry and psychology clinician notes dated up to 5 years before and after surgery. For preoperative scores, notes that were closest to the surgery date were assessed, whereas for posttreatment scores, the note furthest from surgery (but still within the established time frame) was assessed. Not every patient was followed by a psychiatrist at Mayo Clinic. Therefore, patients lacking relevant documentation were excluded from the study. If the patient underwent both procedures, only the second procedure was considered. If the patient had multiple comorbid psychiatric diagnoses, overall symptom severity was scored. The raters compared their scores and resolved any discrepancies through discussion. In the case of continued disagreement, two experienced, board‐certified child and adolescent psychiatrists (J.L.V.V., P.E.C.) assisted with a consensus decision.

### 3.3 Outcome variables

The outcomes were CGI‐I psychiatric symptoms, CGI‐I neurologic symptoms, CGI‐S psychiatric symptoms, and CGI‐S neurologic symptoms. CGI‐I was measured after DBS or VNS treatment, whereas CGI‐S was measured before neuromodulator device placement and after treatment.

### 3.4 Independent variable and covariates

The primary independent variable was treatment with either DBS or VNS. Age (years), sex, preoperative CGI‐S score (for the CGI‐I model), and CGI‐S as a time‐varying covariate were chosen a priori as covariates in the models to increase precision when assessing the effect of treatment on outcomes.

### 3.5 Statistical analysis

Demographic and clinical characteristics were expressed with the sample mean and standard deviation for continuous variables and the frequency and percentage for categorical variables. Two‐sample *t*‐tests (independent samples) with the Satterthwaite method for unequal variances (continuous variables) or the Fisher exact test (categorical variables) were used to identify any variations in patient characteristics between groups.

The difference between treatment groups for CGI‐S scores over time was assessed with a linear mixed model for repeated measures. The mixed model included fixed‐effects terms for group (VNS vs. DBS), time, and group × time interaction. Age, sex, time‐varying CGI‐S psychiatric score (for the CGI‐S neurologic outcome), and time‐varying CGI‐S neurologic score (for the CGI‐S psychiatric outcome) were included as covariates in their respective models. Restricted maximum likelihood estimation, robust standard errors (sandwich or empirical estimator), and type 3 tests of fixed effects were applied, and the Kenward–Roger correction was applied to the compound symmetry covariance structure (Kenward & Roger, 1997). Least‐squares means (LSMs) (adjusted group means) were estimated as part of the mixed model to interpret the group effect (LSM difference between groups). Simple group effects for each time period (preoperatively and after treatment), as well as within‐group changes in CGI‐S after treatment, were also assessed. Cohen d was calculated and interpreted as the effect size estimator.

To examine treatment group differences, we used linear fixed‐effects analysis of covariance (ANCOVA) on each posttreatment CGI‐I outcome measure while controlling for age, sex, and preoperative CGI‐S scores. Separate ANCOVA models were used for each of the two CGI‐I outcome measures. LSMs and robust standard errors (sandwich or empirical estimator) were estimated as part of the linear ANCOVA model. Cohen d was calculated and interpreted as the effect size estimator for the between‐patient treatment group effect.

Statistical analyses were conducted with SAS software, version 9.4 (SAS Institute, Inc.). The (two‐tailed) level of significance was set at *α* = .05, and we implemented the false discovery rate procedure to control for false‐positive results over the multiple tests (Benjamini & Hochberg, 1995).

## 4 RESULTS

### 4.1 Participant characteristics

We identified 138 patients who had DBS or VNS devices implanted to treat neurologic conditions. Of these, 73 patients had comorbid psychiatric diagnoses and were included in the study (DBS, *n* = 23; VNS, *n* = 50). Fifty‐one patients (70%) were male, and the mean (SD) age was 13.9 (5.7) years (range, 4–22 years). A majority of those receiving VNS (*n* = 44, 92%) were diagnosed with intractable seizure disorder. The other patients (*n* = 8, 8%) were diagnosed with Lennox–Gastaut syndrome. Patients in the group receiving DBS (*n* = 15, 65%) had movement disorders or intractable seizure disorder (*n* = 6, 26%). Complications from the surgical procedure were reported for 32 patients (44%). Mean (SD) CGI‐S scores before treatment were 4.47 (1.26) for psychiatric symptoms and 5.64 (0.69) for neurologic symptoms. Patient demographic and clinical characteristics are shown in Table 1.

**TABLE 1:** Patient demographic and clinical characteristics (*N* = 73).

Characteristic	Overall sample (*N* = 73)	Vagal nerve stimulation (*n* = 50)	Deep brain stimulation (*n* = 23)	*p* Value (FDR)^a^
Age, mean (SD), year	13.9 (5.7)	14.1 (6.0)	13.6 (4.9)	.71 (.884)
Female sex, No. (%)^b^	22 (31)	18 (37)	4 (18)	.17 (.872)
Preoperative CGI‐S score, mean (SD)				
Neurologic	5.64 (0.69)	5.60 (0.60)	5.74 (0.86)	.49 (.884)
Psychiatric^b^	4.47 (1.26)	4.27 (1.25)	4.91 (1.19)	.048 (.528)
Comorbid disorder, No. (%)				
Disruptive behavior disorder	10 (13.7)	6 (12.0)	4 (17.4)	.72 (.884)
Neurodevelopmental disorder	56 (76.7)	39 (78.0)	17 (73.9)	.77 (.884)
Attention‐deficit/hyperactivity disorder	18 (24.7)	12 (24.0)	6 (26.1)	.85 (.884)
Anxiety disorder^b^	20 (27.8)	13 (26.5)	7 (30.4)	.78 (.884)
Depression^b^	17 (23.6)	14 (28.0)	3 (13.6)	.24 (.872)
Suicidality^b^	7 (9.9)	5 (10.2)	2 (9.1)	.88 (.884)
Complications from procedure, No. (%)	32 (43.8)	23 (46.0)	9 (39.1)	.62 (.884)

Abbreviations: CGI‐S, Clinical Global Impression scale for illness severity; FDR, false‐discovery rate.

^a^ Two‐sample (independent sample) *t*‐test with the Satterthwaite method for unequal variances (continuous variables) or the Fisher exact test (categorical variables) were used to identify differences between groups. *p* Values (2‐tailed) pertain to comparison of VNS and DBS groups.

^b^ There were two missing observations for sex (one in DBS, one in VNS); three missing observations for preoperative CGI‐S psychiatric score (one in DBS, two in VNS); one missing observation for anxiety (VNS); one missing observation for depression (DBS); and two missing observations for suicidality (one in DBS, one in VNS).

### 4.2 CGI‐S neurologic symptoms

**FIGURE 1:**
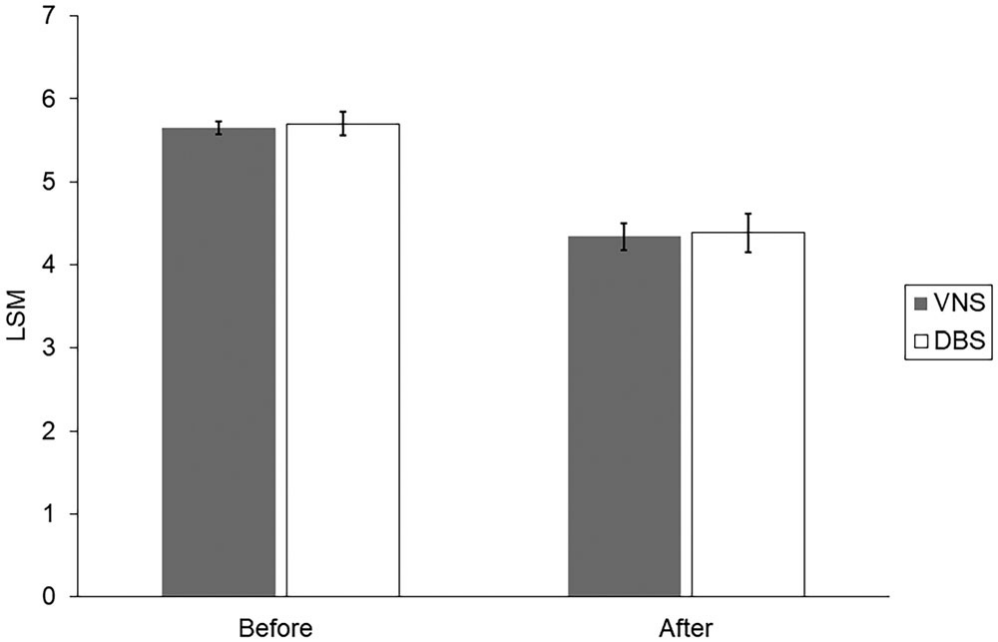
Adjusted least‐squares mean (LSM) for Clinical Global Impression scale for illness severity (CGI‐S) neurologic scores. Between‐group differences were assessed with a linear mixed model for repeated measures. LSM values were adjusted for age, sex, and time‐varying CGI‐S psychiatric scores. No significant differences between groups were observed preoperatively (*p *= .79) or after treatment (*p *= .87). Neurologic symptoms improved after treatment for both groups (*p* < .001). A higher CGI‐S score indicates worse symptoms. Error bars show the standard error. CGI‐S indicates the Clinical Global Impression scale for illness severity; DBS, deep brain stimulation; VNS, vagal nerve stimulation.

The mixed model repeated‐measures analysis did not identify a significant group × time interaction effect (*F* = .01; degrees of freedom [df] = 1,65; *p *> .99). Although we did not observe a significant main effect for group (*F* = .06; df = 1,64; *p *= .81), we did note a significant time effect (*F* = 102.75; df = 1,65; *p *< .001). The LSMs (adjusted CGI‐S neurologic scores) were not significantly different between the two treatment groups preoperatively or posttreatment (Table 2, Figure 1). However, the pattern of the LSMs (adjusted CGI‐S neurologic scores) showed significantly improved neurologic symptoms. Those who received VNS had a mean decrease of 30.3% (*p *< .001; *d* = 1.595), and those who received DBS had a mean decrease of 30.1% (*p *< .001; *d* = 1.529).

**TABLE 2:** Effect of vagal nerve stimulation (VNS) versus deep brain stimulation (DBS) on Clinical Global Impression scale for illness severity (CGI‐S) neurologic and psychiatric scores (mixed model).

	Preoperative					Posttreatment					Difference in CGI‐S				
Outcome and group	LSM (SE)^a^	95% CI of LSM	*F* statistic	*p* Value^b^	Cohen *d*	LSM (SE)^a^	95% CI of LSM	*F* statistic	*p* Value^b^	Cohen *d*	LSM (SE)^a^	95% CI of LSM	*F* statistic	*p* Value^b^	Cohen *d*
**CGI‐S neurologic** ^c^															
VNS	5.650 (.078)	5.493–5.808	NA	NA	NA	4.335 (.161)	4.011–4.658	NA	NA	NA	−1.315 (.143)	−1.601 to −1.030	*F* (1.65) = 84.84	<.001	1.595
DBS	5.698 (.147)	5.403–5.993	NA	NA	NA	4.381 (.235)	3.910–4.851	NA	NA	NA	−1.317 (.219)	−1.755 to −0.878	*F* (1.65) = 35.94	<.001	1.529
LSM group difference^d^	−0.047 (.172)	−.392 to 0.297	*F*(1,65) = .08	.79	.067	−0.046 (.289)	−.624 to 0.532	*F* (1.65) = .03	.87	.039	NA	NA	NA	NA	NA
**CGI‐S psychiatric** ^c^															
VNS	4.120 (.186)	3.748–4.492	NA	NA	NA	4.700 (.214)	4.272–5.128	NA	NA	NA	0.580 (.212)	.156–1.003	*F* (1.65) = 7.46	.008	.667
DBS	4.727 (.266)	4.195–5.259	NA	NA	NA	4.720 (.261)	4.198–5.243	NA	NA	NA	−0.007 (.197)	−.400 to 0.386	*F* (1.65) = .01	.97	.008
LSM group difference^d^	−0.607 (.317)	−1.241 to 0.026	*F* (1.65) = 3.66	.06	.467	−0.020 (.324)	−.669 to 0.628	*F*(1,65) = .01	.95	.015	NA	NA	NA	NA	NA

Abbreviations: LSM, least‐squares mean; NA, not applicable; SE, standard error.

^a^ LSM estimates were adjusted for age, sex, time‐varying CGI‐S psychiatric score (for the CGI‐S neurologic outcome), and time‐varying CGI‐S neurologic score (for the CGI‐S psychiatric outcome).

^b^ *p* Values are associated with the test (*F* statistic) of the difference in LSM estimates or the difference between preoperative and posttreatment CGI‐S scores within each group.

^c^ Higher CGI‐S scores indicate worse symptoms.

^d^ Difference in LSM estimates (VNS vs. DBS).

### 4.3 CGI‐S psychiatric symptoms

**FIGURE 2:**
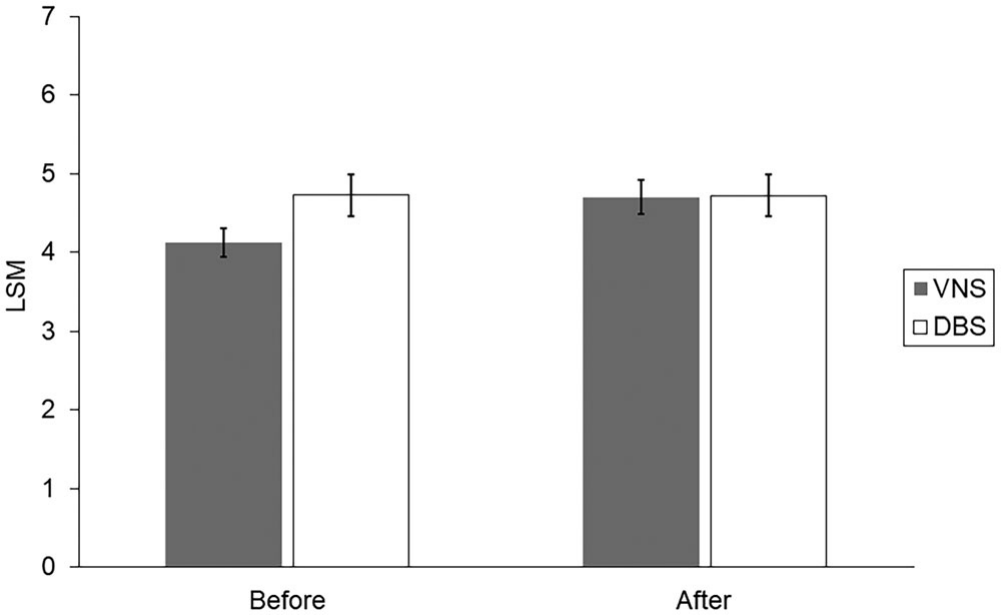
Adjusted least‐squares mean (LSM) for Clinical Global Impression scale for illness severity (CGI‐S) psychiatric scores. Between‐group differences were assessed with a linear mixed model for repeated measures. LSM values were adjusted for age, sex, and time‐varying CGI‐S neurologic scores. No significant differences between groups were observed preoperatively (*p *= .06) or after treatment (*p *= .95). No significant improvement (decrease) was observed for psychiatric symptoms after DBS treatment (*p *= .97). However, patients receiving VNS had a significant worsening effect (increase) (*p *= .008). A higher CGI‐S score indicates worse symptoms. Error bars show the standard error. CGI‐S indicates the Clinical Global Impression scale for illness severity; DBS, deep brain stimulation; VNS, vagal nerve stimulation.

The mixed model repeated‐measures analysis did not identify significant effects for group (*F* = 1.09; df = 1,64; *p *= .30) or time (*F* = 2.84; df = 1,65; *p *= .10), but it did show a significant group × time interaction effect (*F* = 6.61; df = 1,65; *p *= .01). The LSMs (adjusted CGI‐S psychiatric scores) were not significantly different between the two treatment groups preoperatively or posttreatment (Table 2, Figure 2). However, the pattern of the LSMs (adjusted CGI‐S psychiatric scores) showed significantly worsened psychiatric symptoms for some patients. Those who received VNS had a mean increase of 14.1% (*p *= .008; *d* = .667), whereas patients who received DBS had a nonsignificant decrease in symptoms (mean decrease, 0.1%; *p *= .97; *d* = .008).

### 4.4 Illness improvement

The ANCOVA showed no significant treatment group effect on CGI‐I neurologic symptoms (*F* = .06; df = 1,62; *p *= .81) or on CGI‐I psychiatric symptoms (*F* = 2.80; df = 1,62; *p *= .10). The LSMs for the groups (adjusted CGI‐I scores) were not significantly different between the two treatment groups for CGI‐I neurologic symptoms or psychiatric symptoms (Table 3).

**TABLE 3:** Effect of vagal nerve stimulation (VNS) versus deep brain stimulation (DBS) on Clinical Global Impression scale for illness improvement (CGI‐I) neurologic and psychiatric scores analysis of covariance (ANCOVA).

Outcome and group	LSM (SE)^a^	95% CI of LSM	*F* statistic	*p* Value^b^	Cohen *d*
CGI‐I neurologic^c^					
VNS	2.767 (.130)	2.506–3.028	NA	NA	NA
DBS	2.710 (.188)	2.333–3.088	NA	NA	NA
LSM group difference^d^	0.057 (.233)	−.410 to 0.524	*F* (1.62) = .06	.81	.061
CGI‐I psychiatric^c^					
VNS	4.132 (.136)	3.860–4.404	NA	NA	NA
DBS	3.751 (.188)	3.374–4.128	NA	NA	NA
LSM group difference^d^	0.381 (.227)	−.074 to 0.836	*F* (1.62) = 2.80	.10	.418

Abbreviations: CGI‐S, Clinical Global Impression scale for illness severity; LSM, least‐squares mean; NA, not applicable; SE, standard error.

^a^ LSM estimates were adjusted for age, sex, and pretreatment CGI‐S scores.

^b^ *p* Values are associated with the test (*F* statistic) of the LSM group difference.

^c^ Higher CGI‐I score indicates worse symptoms.

^d^ Difference in LSM estimates (VNS vs. DBS).

## 5 DISCUSSION

This naturalistic, retrospective study examined the neurologic and psychiatric outcomes of VNS and DBS for children, adolescents, and young adults. Patients who underwent these procedures had improved neurologic outcomes, with both VNS and DBS showing similar levels of effectiveness for primary neurologic diagnoses. Patients with VNS had a 30.3% reduction in CGI‐S scores, whereas patients with DBS had a 30.1% reduction. Although the neurologic outcomes were comparable, psychiatric outcomes were different among groups. Patients with VNS had a 14.1% increase in CGI‐S scores, indicating worsening clinical outcomes, but the DBS group did not show significant changes within the same time frame. We examined the efficacy of VNS and DBS procedures by comparing CGI‐I scores for psychiatric and neurologic symptoms and noted no significant difference between the groups. Finally, we examined complications associated with the surgical procedures and did not observe any significant differences.

VNS is used to treat intractable seizure disorders, and DBS is limited to treating movement disorders in children and adolescents. Previous research has shown that children receiving VNS achieved marked seizure reduction (>50%), whereas DBS had superior efficacy for treating primary generalized dystonia (Coubes et al., 2000; Isaias et al., 2008; Koy & Timmermann, 2017; Marks et al., 2009; Vasques et al., 2009). Consistent with the previous studies and clinical application of these procedures, our results showed improvement in neurologic symptom severity. However, posttreatment CGI‐S scores showed that patients still had moderate symptoms. The average symptom severity score for both treatment groups was above 4 (Figure 1), indicating that although these procedures were helpful and improved the patients’ quality of life, neither were associated with remission.

Given the retrospective, naturalistic approach of the study, it is difficult to draw definitive conclusions. However, the present findings suggest that young patients who underwent VNS (most of whom had intractable seizure disorders) had worsening psychiatric symptoms. This change is consistent with the forced normalization phenomenon, in which better seizure control and stabilization of epileptiform activity are accompanied by an increase in psychiatric symptoms (Calle‐Lopez et al., 2019). The lack of change in symptoms among those receiving DBS may be because most patients in that group had movement disorders, which may not be associated with the forced normalization phenomenon. Overall, our findings provide indirect evidence of the forced normalization phenomenon for patients with seizure disorders in the context of invasive neuromodulation.

Previous studies have shown psychiatric improvements among patients receiving DBS, such as a reduction in depression symptoms (Lozano et al., 2008, 2012; Mayberg et al., 2005). We did not identify a similar effect, even though most patients in the DBS group had multiple psychiatric comorbidities. The patient cohort was complex and heterogenous because we included patients with movement or seizure disorders. Various interactions between diagnoses, comorbidities, and developmental stages may exist, and they may influence the effectiveness of different methods of stimulation.

We observed no difference in improvement between patients receiving DBS and patients receiving VNS with regard to neurologic and psychiatric symptoms. This finding contrasts with previous research showing that DBS was more effective for neurologic and psychiatric symptoms (Wong et al., 2019; Zhu et al., 2021). We note that our retrospective review has inherent limitations and did not control for many variables that were considered in previous clinical trials. However, VNS may also have increased efficacy in younger populations, making it equally effective as DBS. Given that the patients treated with VNS had epilepsy, forced normalization with respect to psychiatric symptoms may be a consideration for invasive neuromodulation teams to anticipate, monitor, and address in clinical practice.

The placement of intracranial electrodes in DBS is a somewhat more invasive procedure than the implantation of a VNS device. However, implanting VNS is also considered moderately invasive due to lead wire wrapped around the vagus nerve in addition to the subcutaneously placed stimulator. We did not observe any differences in surgical complications between the two groups. Although the rate of complication was high compared to safety reviews for DBS and VNS procedures (Koy et al., 2023; Revesz et al., 2016), it is possible that including all physical complications after the procedure increased the rate of overall complications reported in our study.

## 6 CONCLUSION

Invasive neuromodulation techniques in young patients with neurologic disorders and psychiatric comorbidities merit further prospective, rigorous study. Child and adolescent psychiatrists should take a more active role in the assessment and care of these patients, and they should be members of care teams. Findings from the current study will inform future prospective trials of invasive neuromodulation for children, adolescents, and young adults. Future work should focus on prospective studies designed to assess and monitor psychiatric symptoms and outcomes in young patients undergoing VNS and DBS.

## AUTHOR CONTRIBUTIONS

*Data curation; writing‐original draft; writing‐review editing*: Deniz Yuruk, Can Ozger, and Juan F. Garzon. *Conceptualization; data analyses; data interpretation; writing original draft; writing‐review editing*: Paul A. Nakonezny. *Conceptualization; supervision; data interpretation; writing‐review editing*: Jennifer L. Vande Voort. *Conceptualization; supervision; data curation; data interpretation; writing original draft; writing‐review editing*: Paul E. Croarkin. All authors revised the final manuscript for intellectual contribution and approved the final version. All authors had full access to the data used in the study and accepted responsibility for submitting the paper for publication.

## CONFLICT OF INTEREST STATEMENT

Dr Croarkin has received research grant support from Neuronetics Inc, NeoSync Inc, and Pfizer Inc. He has received in‐kind support (equipment, supplies, and genotyping) for research studies from Assurex Health Inc., Neuronetics Inc., and MagVenture Inc. He has consulted for Engrail Therapeutics Inc., Myriad Neuroscience, Procter and Gamble, and Sunovion. Dr Vande Voort has received in‐kind support (supplies and genotyping) from Assurex Health Inc. The other authors declare that they have no conflicts of interest.

### PEER REVIEW

The peer review history for this article is available at https://publons.com/publon/10.1002/brb3.3452.

## PATIENT CONSENT STATEMENT

All patients completed research authorization forms, giving permission to use medical records for research.

## Data Availability

The data set used and analyzed for this study is available from the corresponding author upon reasonable request.
